# The p75 Neurotrophin Receptor Facilitates TrkB Signaling and Function in Rat Hippocampal Neurons

**DOI:** 10.3389/fncel.2019.00485

**Published:** 2019-10-29

**Authors:** Juan P. Zanin, Laura E. Montroull, Marta Volosin, Wilma J. Friedman

**Affiliations:** Department of Biological Sciences, Rutgers University, Newark, NJ, United States

**Keywords:** TrkB, p75 neurotrophin receptor, brain derived neurotrophic factor, Akt, Erk

## Abstract

Neurotrophins activate Trk receptor signaling to support neuronal survival and many aspects of neuronal function. Early studies demonstrated that TrkA formed a complex with the p75 neurotrophin receptor (p75^*NTR*^), which increased the affinity and selectivity of NGF binding, however, whether interaction of p75^*NTR*^ with other Trk receptors performs a similar function to enhance ligand binding has not been demonstrated. We investigated the interaction of TrkB with full length p75^*NTR*^ in hippocampal neurons in response to BDNF and found that the association of these receptors occurs after ligand binding and requires phosphorylation of TrkB, indicating that formation of this receptor complex was not necessary for ligand binding. Moreover, the interaction of these receptors required internalization and localization to early endosomes. We found that association of TrkB with p75^*NTR*^ was necessary for optimal downstream signaling of the PI_3_K-Akt pathway, but not the Erk pathway, in hippocampal neurons. The absence of p75^*NTR*^ impaired the ability of BDNF to rescue hippocampal neurons in a trophic deprivation model, suggesting that p75^*NTR*^ facilitates the ability of TrkB to activate specific pathways to promote neuronal survival.

## Introduction

The neurotrophin family of trophic factors, which includes NGF, BDNF, NT3, and NT4, regulate multiple aspects of neuronal survival and function by interacting with distinct receptor complexes. These factors promote survival, axonal growth, and synaptic activity by signaling via Trk receptors, and can induce apoptosis by signaling via the p75 neurotrophin receptor (p75^*NTR*^). The p75^*NTR*^ has been shown to associate with a variety of co-receptors, such as TrkA or sortilin, to facilitate binding to NGF (Hempstead et al., 1991) or proNGF (Nykjaer et al., 2004), respectively. Previous studies demonstrated that the association of p75^*NTR*^ with TrkA increased the affinity and selectivity of NGF binding, promoting TrkA signaling and supporting survival and differentiation of sympathetic neurons (Hempstead et al., 1991). In contrast, the association of p75^*NTR*^ with a member of the sortilin family allows the binding of proneurotrophins and apoptotic signaling by p75^*NTR*^ (Lee et al., 2001; Volosin et al., 2006). Once receptors bind their ligands, receptor internalization and trafficking are important aspects of their signaling. Several studies have focused on retrograde trafficking of neurotrophin receptors from the axon terminal to the soma (Ginty and Segal, 2002; Schmieg et al., 2014), however, the route of endosomal trafficking within the soma can determine which signaling pathways are activated and the duration of signaling. Moreover, localization of neurotrophin receptors may be different depending on cell type (Yano and Chao, 2005), and Trk receptors localized to different intracellular locations have distinct functions. TrkB receptors at the synapse can promote glutamatergic signaling and modulate synaptic activity (Schinder and Poo, 2000) while TrkB receptors in dendrites can promote BDNF-induced branching (Lazo et al., 2013).

Several studies have evaluated the trafficking of Trk receptors and p75^*NTR*^ independently in a variety of neuronal cell types (Bronfman et al., 2003; Chen et al., 2005; Hibbert et al., 2006; Lazo et al., 2013; Escudero et al., 2014). Previous studies on the trafficking of p75^*NTR*^ in PC12 cells and sympathetic neurons demonstrated that BDNF, a ligand that binds only to p75^*NTR*^ in these cells, elicited internalization and retrograde transport in compartments independent of Trk signaling (Hibbert et al., 2006). Additionally, p75^*NTR*^ can be internalized in Rab5-positive early endosomes, trafficked to multivesicular bodies, and released from the cells in exosomes (Bronfman et al., 2003; Escudero et al., 2014).

Studies investigating trafficking of Trk receptors in PC12 cells showed that TrkA and TrkB were trafficked differently in response to their respective ligands, NGF, or BDNF. NGF induced TrkA to be recycled to the plasma membrane, while BDNF elicited TrkB trafficking to the lysosome for degradation (Chen et al., 2005). This difference was reported to be due to a specific sequence in the TrkA juxtamembrane domain that was absent from TrkB. However, in hippocampal neurons, BDNF was shown to induce the localization of TrkB to rab11-positive recycling endosomes to promote dendritic branching (Lazo et al., 2013) rather than to the lysosome, indicating that ligand-induced trafficking may differ in distinct cell types.

In hippocampal neurons, treatment with BDNF elicits an association between TrkB and full length p75^*NTR*^. Whether these receptors form a complex that is maintained during internalization and trafficking, and whether subcellular localization and signaling is altered by the association of the two receptors, is unknown. Since specific subcellular localization of signaling proteins is critical for their function, we have analyzed whether BDNF-induced trafficking and signaling of TrkB is altered in the absence of p75^*NTR*^.

## Materials and Methods

### Neuronal Cultures

All animal studies were conducted using the National Institutes of Health guidelines for the ethical treatment of animals with approval of the Rutgers IACUC. Pregnant rats were sacrificed by exposure to CO2 and soaked in 70% ethanol for 5 min. Rats lacking p75^*NTR*^ were obtained from SAGE/Horizon Laboratories and confirmed by us using PCR, Western blot, and immunostaining to be lacking p75^*NTR*^. Rat fetuses were removed at embryonic day 18 (E18) under sterile conditions and kept in PBS on ice. The hippocampus was dissected, dissociated by trituration in serum-free medium, plated on polylysine (0.1 mg/ml) coated tissue culture wells or glass coverslips, and maintained in a serum-free environment (Friedman et al., 1993; Farinelli et al., 1998). Medium consists of a 1:1 mixture of Eagle’s MEM and Ham’s F12 supplemented with glucose (6 mg/ml), putrescine (60 μM), progesterone (20 nM), transferrin (100 μg/ml), selenium (30 nM), penicillin (0.5 U/ml), and streptomycin (0.5 μg/ml). In all experiments, neurons were cultured for 4–5 days before treatment. Cultures maintained under these conditions contained <2% glial cells, confirmed by staining for glial markers.

### Immunoprecipitation and Western Blot Analysis

Cultured hippocampal neurons were treated with BDNF (25 ng/ml) for different time points and compared with untreated control neurons. Cells were lysed in a buffer consisting of Tris–buffered saline with 10% Triton, 0.6 M octylglucoside, and protease inhibitor cocktail (Roche, 11836153001) and phosphatase inhibitor cocktail (Roche, 04906845001). Total protein was quantified by the Bradford assay (Bio-Rad, Hercules, CA, United States). Samples were equilibrated to have the same total protein quantity and the same final volume. Lysates were subjected to Western Blot analysis with antibodies to pAkt (Ser473, Cell Signaling, 587F11) and pErk1/2 (Thr202/Tyr 204, Cell Signaling, 9106). Blots were re-probed for total Akt (Cell Signaling, 9272) and Erk1/2 (Cell Signaling, 9102). For the immunoprecipitation analysis, lysates were precleared with 5 μl of protein G-magnetic beads (New England Bio Labs S1430S) at 4°C for 60 min. For p75 immunoprecipitation, 150–200 μg total protein from hippocampal neuron cleared lysates was incubated with anti-p75 (Millipore, MAB365, RRID: AB_2152788) overnight on a rocking platform at 4°C. Protein G-magnetic beads (10 μl per 100 μg of total protein) was then added to the lysates and kept for an additional 2 h at 4°C. Immunoprecipitates were collected using a magnetic rack (New England Bio Labs S1506S) and washed 3X with lysis buffer and eluted by adding 30 μl of loading buffer and subjected to Western blot analysis with antibodies to TrkB (Millipore, 07-225, RRID: AB 310445). Blots were stripped and re-probed with anti-p75 (Millipore, MAB365, RRID: AB_2152788). All Western blot analyses were performed at least four times with samples from independent experiments. Membranes were visualized using either ECL (Pierce) or scanned with the Odyssey infrared imaging system (LI-COR Bioscience). To ensure equal protein levels in Western blots, membranes were stained with Ponceau and re-probed with anti-actin (Sigma, A5316, RRID: AB 476743).

### Immunocytochemistry

Cultured cells were treated with BDNF for the indicated times, washed with PBS and fixed in 4% paraformaldehyde for 15 min at room temperature. Cells were permeabilized with PBS/0.5%Triton X-100 and then blocked for 1 hr with PBS/1% BSA/5% normal goat serum and exposed to primary antibodies overnight at 4°C. Cells were washed 3X with PBS, and exposed to secondary antibodies coupled to different fluorophores for 1 hr at room temp. Cells were washed three times in PBS and then mounted using Prolong Gold containing 4′,6′-diamidino-2-phenylindole (DAPI) (Life Technologies P36934). Cells were analyzed by epifluorescence (Nikon Eclipse TE200), confocal (Zeiss 510 Meta), or enhanced resolution (Leica SP8, 63X, 1.4 NA, Lightning Mode) microscopy. No immunostaining was seen in controls with omission of the primary antibodies.

### FRET Analysis

The interaction of TrkB with p75^*NTR*^ was analyzed by acceptor photobleaching FRET. Briefly, FRET occurs because the acceptor receives parts of the energy emitted by the donor molecule. Therefore, when the acceptor is bleached, the donor emission will increase. This is only possible when the two molecules analyzed are close enough to induce the energy transfer. We used Alexa 488 (Life Technologies, A-11039, A32790 or A-11015) as the energy donor and Alexa 555 (Life Technologies, A-31570 or A-21432) as the acceptor. Images were acquired before and after the acceptor was bleached. The fluorophore was bleached using the laser 549 at 100% power for 200 iterations with the 63X oil objective of a Zeiss LSM 510 Meta microscope. Only the cells with a reduction of at least 80% in the intensity of the acceptor channel were used for the analysis. The donor fluorescence intensity was quantified before and after bleaching the acceptor fluorophore. The ΔF/F formula (Fluorescence after – Fluorescence Before/Fluorescence Before) was used to measure the change in the intensity. The average of the ΔF/F was used for statistical analysis. In the experiments for early endosome marker, the analysis of fluorescence intensity was done in vesicle positives for Rab 5 that contain p75NTR and TrkB.

### Receptor Biotinylation

Hippocampal neurons were maintained for 5 d before treatment. To identify proteins internalized after treatment, cells were rinsed twice with PBS containing 1 mM CaCl_2_ and 0.5 mM MgCl_2_ (PBS-Ca-Mg) and then incubated with 1mg/ml Sulfo-NHS-SS-Biotin dissolved in biotinylation buffer (0.01 m TEA, pH 7.4, 2 mm CaCl_2_, 150 mm NaCl) for 1 h at 4°C. Remaining biotin was then quenched with PBS-Ca-Mg containing 0.1M glycine for 20 min at 4°C. After warming to 37°C, cells were treated with 25 ng/ml BDNF, NGF, or vehicle for the indicated times. As a control for internalization, cells treated with BDNF were maintained at 4°C. After treatment, biotin from the proteins that remained in the cell surface was cleaved using glutathione buffer (5M NaCl, 0.5M EDTA, 1% BSA, 1.5% Glutathione, 5N NaOH). Cells were then lysed in RIPA buffer 1% NP-40, 0.1% SDS, 0.1% deoxycholate, 150 mM NaCl, 1 mM EDTA, 10 mM Tris, pH 8.0, protease inhibitor cocktail (Roche, 11836153001) and phosphatase inhibitor cocktail (Roche, 04906845001) and incubated on ice for 30 min. Internalized biotinylated proteins were isolated using streptavidin-conjugated sepharose beads (Pierce), eluted from the beads, separated by SDS-PAGE, and immunoblotted with the corresponding antibodies.

To determine interactions of internalized receptors, hippocampal neurons were incubated with or without BDNF for 15 or 30 min to induce receptor internalization prior to biotinylation. The remaining cell-surface proteins were biotinylated with 1mg/ml Sulfo-NHS-SS-Biotin for 30 min at 4^*o*^C. After quenching the excess biotin with 0.1 M glycine, cells were harvested with RIPA lysis buffer. Lysates were incubated with Streptavidin-beads overnight at 4°C to remove proteins remaining on the cell surface. The supernatants of the streptavidin-beads, representing the internalized proteins from control and BDNF treated neurons, were immunoprecipitated with rabbit anti-p75^*NTR*^ (Millipore, MAB365, RRID: AB_2152788) or mouse anti-p75^*NTR*^ (Millipore, MAB365, RRID: AB 2152788) antibodies overnight at 4°C, separated by SDS-PAGE, and immunoblotted for TrkB (Millipore, 07-225, RRID: AB 310445) and p75NTR (Millipore, MAB365, RRID: AB_2152788).

### Survival Assay

On the fourth day in culture, fifty percent of the SFM was replaced by a media lacking insulin (“insulin deprivation” media), with or without BDNF (25 ng/ml). Control cultures did not have media change. One day later, hippocampal neurons from WT or KO rats were lysed and intact nuclei were counted using a hemocytometer to assess cell viability as described before (Friedman, 2010; Greenwood et al., 2018). Cell counts were performed in 3 to 5 independent experiments with triplicate cultures per experiment.

### Statistical Analysis

Quantitative data are presented as mean ± SEM. The results represent the average of at least three independent experiments, unless specifically indicated. Statistical significance was determined by ANOVA followed by Tukey’s test or ANOVA repeated measurements with Sidak’s test with *p* < 0.05 considered significant.

## Results

### BDNF Induces Association Between TrkB and p75NTR That Depends on TrkB Activation

**FIGURE 1:**
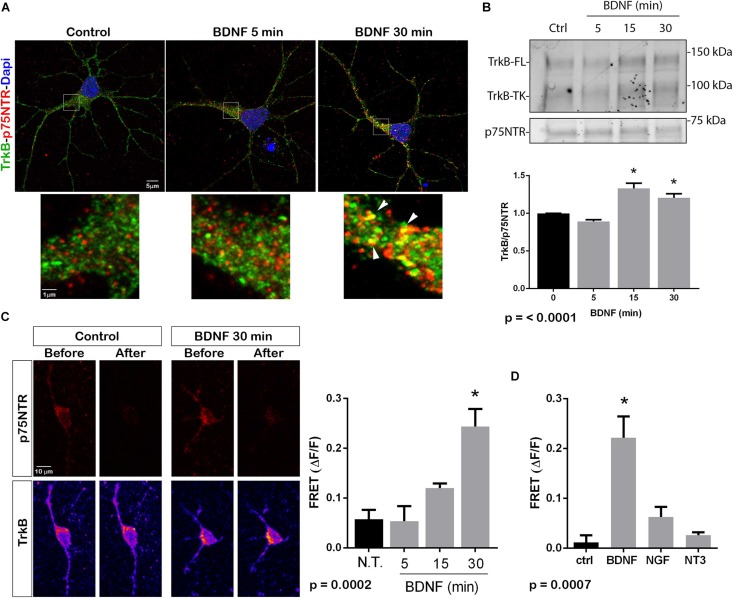
BDNF elicits association between TrkB and p75NTR. **(A)** Cultured hippocampal neurons were treated with BDNF (25 ng/ml) at 37°C for 5 or 30 min. Cells were immunostained with goat anti-p75^*NTR*^ (red) and rabbit anti-trkB (green). Top images show an entire neuron with each treatment, and boxes indicate the areas analyzed by superresolution microscopy (Leica SP8, 63X, and 1.4 NA), which demonstrated the increase in double-labeled puncta indicative of association of p75^*NTR*^ and TrkB. Arrows denote areas of colocalization (yellow puncta). **(B)** Lysates stimulated with BDNF for the indicated times, were immunoprecipitated (IP) with anti-p75^*NTR*^, probed with anti-TrkB, and reprobed with anti-p75^*NTR*^. BDNF stimulation increased the amount of TrkB that coimmunoprecipitated with p75^*NTR*^ in time-dependent manner. Graph shows quantification of 4 independent experiments. ^∗^ indicates *p* < 0.001 by ANOVA with Tukey’s *post hoc* analysis. **(C)** Receptor photobleaching FRET was used to analyze interaction of p75^*NTR*^ and TrkB after BDNF treatment of hippocampal neurons. Cultured neurons were treated with 50 ng/ml BDNF for 5, 15, or 30 min. Cells were immunostained with mouse anti-p75^*NTR*^ (red) and chicken anti-TrkB (green). The acceptor fluorophore was photobleached and the increased fluorescence of the donor fluorophore is indicated in the graph. Images show p75^*NTR*^ (top) and TrkB (bottom) immunostaining before and after photobleaching with and without 30 min BDNF treatment. The graph shows quantification of fluorescence intensity at all time points. ^∗^ indicates *p* = 0.0002 by ANOVA with Tukey’s *post hoc* analysis. **(D)** The specificity of ligand-induced TrkB-p75^*NTR*^ interactions was analyzed by FRET. Hippocampal neurons were treated as indicated for 30 min, and the level of fluorescence was measured after acceptor photobleaching. BDNF, but not NGF or NT3, increased the association of TrkB with p75^*NTR*^. ^∗^ indicates *p* = 0.0007 by ANOVA with Tukey’s *post hoc* analysis.

**FIGURE 2:**
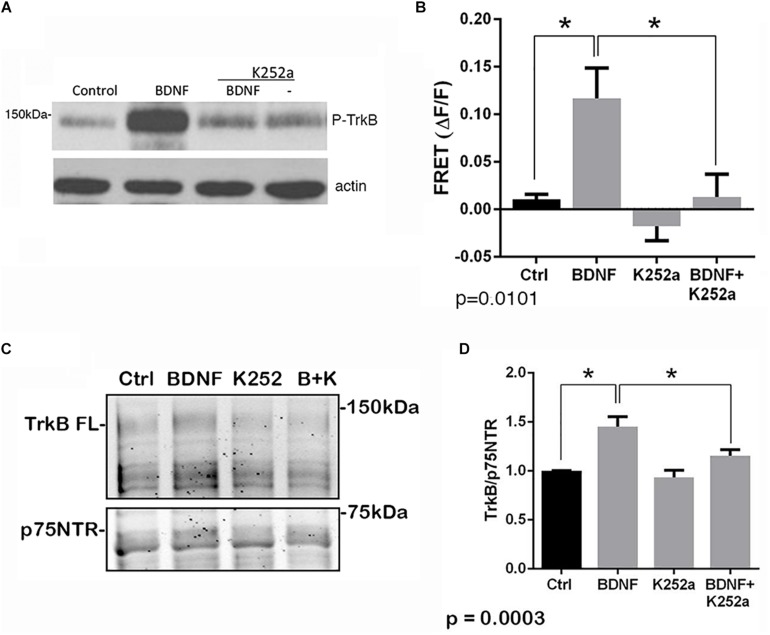
Interaction of TrkB with p75^*NTR*^ requires kinase activation. Hippocampal neurons were incubated with 25 ng/ml BDNF for 30 min with or without pretreatment with 200 nM K252a for 30 min. **(A)** Phosphorylation of TrkB by BDNF was prevented by preincubation with K252a. Total lysates were analyzed by Western blot, using an anti-phospho-Trk antibody. The membrane was reprobed with an anti-actin antibody. **(B)** K252a pretreatment inhibited BDNF-induced p75^*NTR*^-TrkB association. Acceptor photobleaching FRET analysis demonstrated that K252a pretreatment prevented association of p75^*NTR*^ and TrkB. Quantification of 3 independent experiments, ^∗^*p* = 0.0101 by ANOVA with Tukey’s *post hoc* comparison. **(C)** Lysates were immunoprecipitated with anti-p75^*NTR*^ antibody, probed with anti-TrkB, and reprobed with anti-p75^*NTR*^ antibody. **(D)** Quantification of 6 independent experiments, ^∗^*p* = 0.0003 by ANOVA with Tukey’s *post hoc* analysis.

The p75^*NTR*^ can interact with different co-receptors, with distinct consequences for activation of signaling pathways and effects on cellular function. In cultured embryonic hippocampal neurons, BDNF induced an increased association between TrkB and full length p75^*NTR*^. Immunostaining for TrkB and p75^*NTR*^ showed an increase in intracellular colocalization after 30 min of BDNF treatment, shown using enhanced resolution microscopy (Figure 1A). Additionally, co-immunoprecipitation of BDNF-treated neurons showed increased association of p75^*NTR*^ with TrkB after 15 and 30 min (Figure 1B), and FRET analysis after acceptor photobleaching showed increased fluorescence of the donor fluorophore (Figure 1C). The interaction of p75^*NTR*^ with TrkB was seen with BDNF treatment, but not NGF or NT3 treatment, indicating the specificity of the response (Figure 1D). Interestingly, although BDNF is known to rapidly activate TrkB phosphorylation within 5 min (Marsh et al., 1993), the interaction between TrkB and p75^*NTR*^ was minimal after 5 min of BDNF treatment, and was only increased after 15 and 30 min of treatment, indicating that association of TrkB with p75^*NTR*^ occurred after TrkB was activated by the ligand, and suggesting that formation of a TrkB/p75^*NTR*^ complex was not required for BDNF to bind and activate TrkB. Early studies had indicated that p75^*NTR*^ interacted primarily with the phosphorylated form of TrkB (Bibel et al., 1999). To confirm whether TrkB phosphorylation was required for association with p75^*NTR*^, neurons were treated with K252a to prevent BDNF-induced TrkB phosphorylation (Figure 2A). K252a prevented the interaction of TrkB with p75^*NTR*^ assessed by FRET analysis (Figure 2B) and co-immunoprecipitation (Figures 2C,D).

### BDNF Induces TrkB-p75^*NTR*^ Interaction in the Endosomal Pathway

**FIGURE 3:**
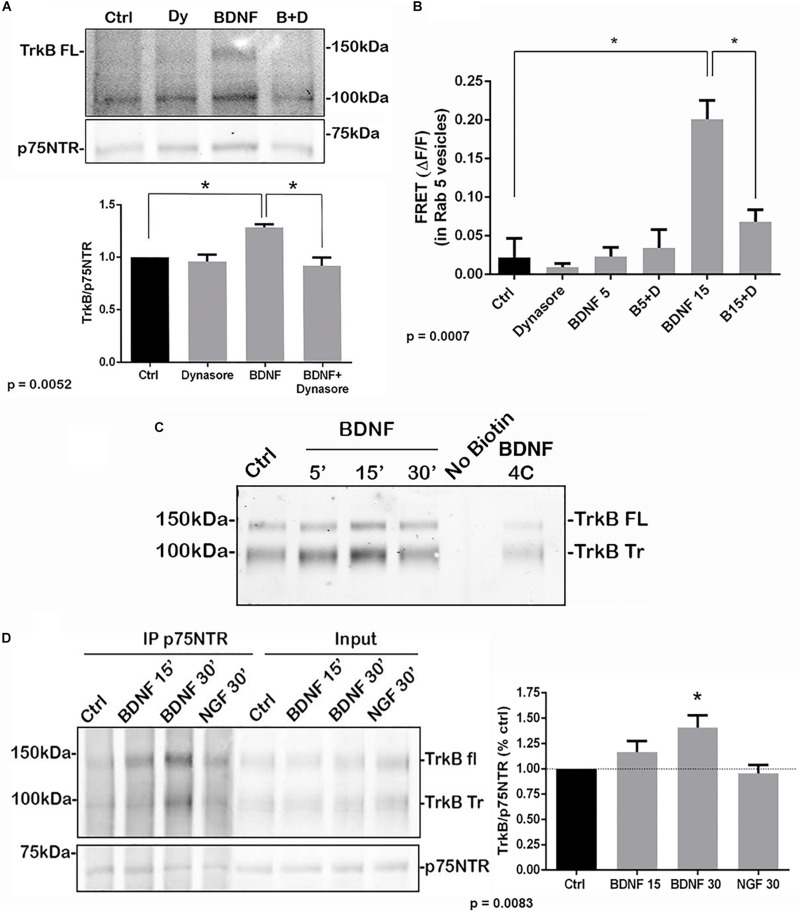
Association of TrkB with p75^*NTR*^ requires internalization. **(A)** Hippocampal neurons were treated with Dynasore for 30 min to prevent internalization prior to treatment with BDNF. Co-IP analysis demonstrated that Dynasore treatment prevented BDNF-induced association of TrkB with p75^*NTR*^. Lysates of hippocampal neurons treated as indicated were immunoprecipitated with anti-p75^*NTR*^ and probed with anti-TrkB. Quantification of 3 independent experiments is shown below. ^∗^ indicates significance at *p* = 0.0052 by ANOVA with Tukey’s *post hoc* analysis. **(B)** FRET analysis also demonstrates that association of TrkB with p75^*NTR*^ induced by 15 min of BDNF treatment was inhibited by Dynasore (B15 + D), quantification of 3 independent experiments, ^∗^*p* = 0.0007 by ANOVA. **(C)** Hippocampal neurons were biotinylated and then treated with BDNF as indicated to induce receptor internalization. BDNF treatment elicited internalization of TrkB after 15 min which was prevented by incubation at 4°C. **(D)** Hippocampal neurons were first treated with BDNF or NGF as indicated to induce receptor internalization. The cells were then biotinylated and streptavidin used to remove the proteins remaining on the cell surface. The internalized proteins were then immunoprecipitated with anti-p75^*NTR*^ and probed for TrkB. The graph shows the increased association of internalized TrkB with p75^*NTR*^ after 30 min of BDNF treatment. ^∗^*p* = 0.0083.

To determine whether internalization of TrkB and p75^*NTR*^ was necessary for the receptors to associate, hippocampal neurons were treated with dynasore, an inhibitor of dynamin GTPase, to prevent dynamin-mediated endocytosis. Dynasore treatment prevented the interaction of TrkB with p75^*NTR*^, analyzed by co-IP (Figure 3A) and FRET (Figure 3B), indicating that internalization was necessary for association of the receptors. Additionally, surface biotinylation experiments were performed. Hippocampal neurons were biotinylated and then treated with BDNF to promote internalization. After 15 min, TrkB was detected following streptavidin pulldown, indicating internalization of the receptor (Figure 3C). An increase in internalized truncated as well as full length TrkB was detected. Cells maintained at 4°C showed no increased receptor internalization with BDNF treatment.

To confirm that the internalized TrkB and p75^*NTR*^ receptors were interacting, hippocampal neurons were first treated with BDNF to promote receptor internalization, then the proteins remaining on the cell surface were biotinylated. Then, cells were lysed and the cell surface proteins were removed by streptavidin pulldown. The remaining supernatants of these pulldowns, representing the internalized proteins, were analyzed by immunoprecipitation with anti-p75^*NTR*^ followed by Western blot for TrkB and p75NTR, and showed increased TrkB association with p75^*NTR*^ at 15 and 30 min after BDNF treatment (Figure 3D). Treatment with NGF as a control did not show interaction of internalized TrkB and p75^*NTR*^.

**FIGURE 4:**
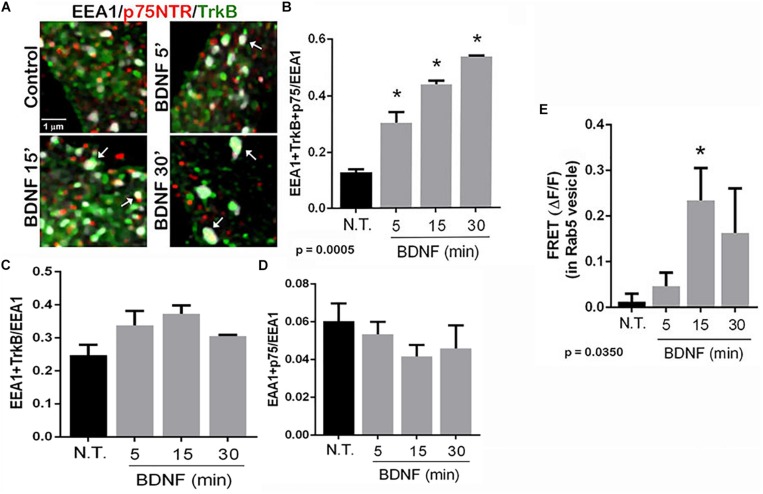
TrkB and p75^*NTR*^ associate in early endosomes. **(A)** Hippocampal neurons were triple-labeled with antibodies to p75^*NTR*^, TrkB, and EEA1 to label early endosomes. Enhanced resolution microscopy analysis shows the increase in triple labeling (arrows) over time with BDNF treatment. **(B)** Quantification of triple labeled puncta, indicating that p75^*NTR*^ and TrkB are colocalized in the early endosomes, *p* = 0.0005 by ANOVA with Tukey’s *post hoc* analysis. **(C)** Quantification of double-labeled puncta for EEA1 and TrkB. **(D)** Quantification of puncta double-labeled for EEA1 and p75NTR. **(E)** Acceptor photobleaching FRET analysis of p75^*NTR*^ and TrkB in early endosomes, quantification of 3 independent experiments, ^∗^*p* ≤ 0.0001 by ANOVA with Tukey’s *post hoc* analysis.

To determine in which intracellular compartments TrkB and p75^*NTR*^ were interacting, triple labeling was performed to assess the localization of the receptors to early endosomes, and labeling was analyzed by enhanced resolution microscopy. The number of triple labeled puncta, representing colocalization of TrkB, p75^*NTR*^ with the early endosome marker EEA1, was quantified with or without BDNF treatment, and showed that BDNF treatment increased localization of both TrkB and p75^*NTR*^ to the early endosomes over time (Figures 4A,B). Analysis of the individual receptors in the EEA1-positive endosome did not show any change with BDNF treatment (Figures 4C,D). Additionally, FRET analysis confirmed an increase in TrkB-p75^*NTR*^ association in the Rab 5-labeled early endosomes following BDNF treatment (Figure 4E).

### p75^*NTR*^ Is Necessary for Optimal TrkB Signaling and Function

**FIGURE 5:**
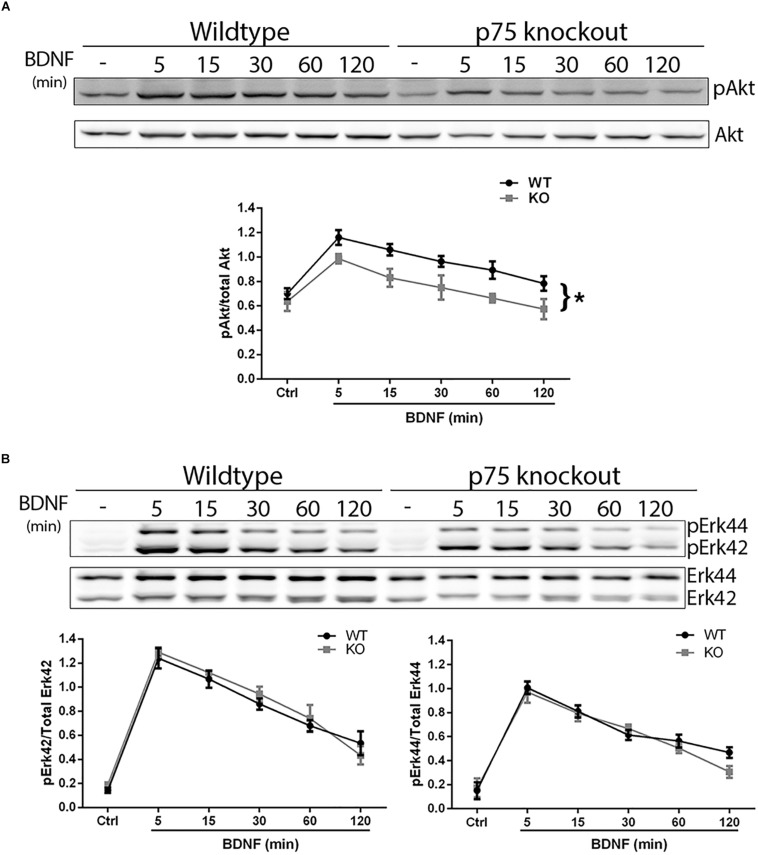
Lack of p75^*NTR*^ attenuates Akt, but not Erk, activation. Hippocampal neurons from WT or p75^*NTR*– /–^ rats were cultured for 5 days and treated with BDNF (25 ng/ml) for the indicated times. **(A)** Lysates were probed for P-Akt and total Akt. Graph indicates the ratio of P-Akt to total Akt, *n* = 3 independent experiments, ^∗^*p* = 0.0405 comparing WT vs. KO by ANOVA repeated measurement. **(B)** Lysates were probed for P-Erk and total Erk. Graph indicates the ratio of P-Erk42 to total Erk42 and P-Erk44 to total Erk44, *n* = 3 independent experiments, *p* = 0.63 for P-Erk42 and *p* = 0.62 for P-Erk44 by ANOVA repeated measurement with Sidak’s *post hoc* analysis.

We investigated whether the absence of p75^*NTR*^ affected the ability of TrkB to signal in response to BDNF. Hippocampal neurons were cultured from WT or p75^*NTR*–/–^ rats. We found that the absence of p75^*NTR*^ attenuated the ability of BDNF to induce and maintain phosphorylation of Akt (Figure 5A). However, phosphorylation of Erk was unaffected by the absence of p75^*NTR*^ (Figure 5B).

**FIGURE 6:**
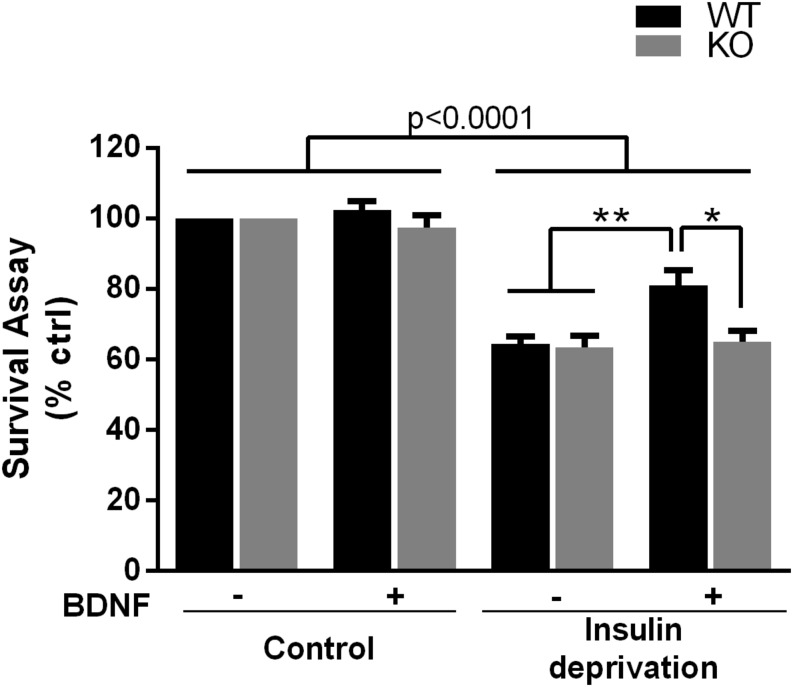
Absence of p75^*NTR*^ impairs rescue of hippocampal neurons by BDNF in a trophic deprivation model with reduced insulin, decreasing survival of the hippocampal neurons by 40%. BDNF (25 ng/ml) provided significant protection to the WT neurons but was unable to rescue the p75^*NTR*– /–^ neurons. ^∗∗^*p* < 0.006; ^∗^*p* = 0.013 by ANOVA repeated measurement with Sidak’s *post hoc* analysis.

The PI_3_K-Akt pathway is critical for signaling neuronal survival, therefore we tested whether survival of hippocampal neurons from the p75^*NTR*–/–^ rats was compromised. Although basal survival of cultured hippocampal neurons from the p75^*NTR*–/–^ rats was not different from WT, we used a trophic withdrawal model of reducing insulin in the media to test the ability of BDNF to rescue the neurons. BDNF (25 ng/ml) was able to rescue WT neurons from insulin depletion, however, BDNF was unable to rescue neurons lacking p75^*NTR*^ (Figure 6), suggesting that p75^*NTR*^ facilitates the ability of TrkB to activate the PI_3_K pathway and promote neuronal survival.

## Discussion

Each of the neurotrophin receptors, the Trk family and p75^*NTR*^, can signal in response to its neurotrophin or proneurotrophin ligand. Trk receptors have been well-established to promote neuronal survival and differentiation when activated by mature neurotrophins. The p75^*NTR*^, not having any kinase activity, recruits different intracellular binding proteins to activate signaling pathways that can have multiple different functions depending on the cellular context. These actions of p75^*NTR*^ depend on the particular ligand and co-receptor that associate with the receptor (Barker, 2004). Interaction of p75^*NTR*^ with a member of the sortilin family can promote apoptosis in response to proneurotrophins (Lee et al., 2001; Ibanez and Simi, 2012). In contrast, early studies demonstrated that association between TrkA and p75^*NTR*^ was required to generate a high affinity binding site for NGF (Hempstead et al., 1991) and that p75^*NTR*^ conferred greater selectivity for Trk receptors to bind their specific ligands (Bibel et al., 1999; Patapoutian and Reichardt, 2001). Moreover, the localization of TrkA was shown to influence the cellular response to NGF, with internalization being required for a differentiation, but not a survival, response in PC12 cells (Zhang et al., 2000). However, the nature and association of p75^*NTR*^ with other Trk receptors has not been thoroughly investigated. Previous studies had shown that p75^*NTR*^ interacts preferentially with the phosphorylated form of TrkB (Bibel et al., 1999), which we confirmed, suggesting that the association of the two receptors occurs after TrkB is activated and thus not required for binding of the ligand. Moreover, a previous study also showed that endocytosis was necessary for TrkA or TrkB-induced activation of Akt, but not Erk (Zheng et al., 2008). We therefore investigated whether association of TrkB with p75^*NTR*^ was required for proper trafficking and signaling.

### Localization and Signaling

It has become clear that localization of receptors and signaling molecules to specific intracellular compartments can regulate the specific pathways activated and modulate the cellular response (Bucci et al., 2014). Previous studies have investigated neurotrophin-induced trafficking of Trk receptors and p75^*NTR*^ independently in a variety of cell types. BDNF treatment of PC12 cells induced trafficking of TrkB to lysosomes (Chen et al., 2005), however, in hippocampal neurons BDNF elicited TrkB localization to Rab5-positive early endosomes (Moya-Alvarado et al., 2018) and Rab11-positive recycling endosomes (Lazo et al., 2013) to modulate dendritic branching, suggesting that the trafficking and signaling may be dependent on the particular cell context. In contrast to TrkB, p75^*NTR*^ did not traffic to lysosomes in PC12 cells and sympathetic neurons, but instead could be found in Rab11-positive endosomes and multivesicular bodies (Escudero et al., 2014). This group also found little p75^*NTR*^ in RAB5+ early endosomes. Our observations indicate that in hippocampal neurons, BDNF induced trafficking of both p75^*NTR*^ and TrkB to Rab5 and EEA1-positive early endosomes, and that localization of TrkB to the early endosomes was reduced in the absence of p75^*NTR*^. BDNF did not elicit any change in the number of EEA1-positive endosomes with either TrkB or p75^*NTR*^ alone. A previous study had indicated that p75^*NTR*^ can interact with Rab5 to regulate glucose uptake in adipocytes (Baeza-Raja et al., 2012), suggesting that p75^*NTR*^ may facilitate internalization of other signaling proteins, such as TrkB, into early endosomes by interacting with Rab5.

TrkB is known to activate both the PI_3_K-Akt and Ras-ERK pathways to regulate and coordinate numerous cellular functions. Localization of these signaling proteins within the cell can impact activation of specific downstream pathways. PI_3_K can phosphorylate phosphoinositides at the plasma membrane or in internal membrane compartments such as early endosomes, and activate Akt locally at those sites (Jethwa et al., 2015). Since our studies demonstrate that TrkB and p75^*NTR*^ interact at the early endosome, it is possible that this localization is required for optimal activation of the Akt pathway. Indeed, our results demonstrate that the absence of p75^*NTR*^ specifically attenuated activation of Akt, but not Erk. Previous studies have suggested that Akt activation by BDNF requires internalization and endosomal localization, and may occur at the early endosome (Zahavi et al., 2018), and this is supported by our current study.

The p75^*NTR*^ can interact with numerous co-receptors in response to different ligands, and can influence a variety of cellular processes (Barker, 2004). In specific circumstances, p75^*NTR*^ has been shown to undergo intramembrane proteolysis by alpha secretase to shed the extracellular domain, generating a C-terminal fragment (CTF), which then can be cleaved to generate the intracellular domain (ICD). The cleavage of p75^*NTR*^ is essential for its role in promoting neuronal apoptosis (Coulson et al., 2000; Volosin et al., 2008), however, whether p75^*NTR*^ cleavage is necessary for other receptor associations and functions is unclear. Studies investigating the interaction of p75^*NTR*^ with TrkA have yielded conflicting results, with one study demonstrating that TrkA interacts with either the full-length p75NTR or the CTF, but that generation of the ICD abrogates that interaction (Jung et al., 2003), while other studies suggested that the p75^*NTR*^ ICD interacts with TrkA and potentiates NGF binding (Ceni et al., 2010; Kommaddi et al., 2011; Matusica et al., 2013). It is also unclear whether TrkA and TrkB show the same interactions with p75^*NTR*^. There are differences in the intracellular domains of TrkA and TrkB that influence their intracellular signaling and trafficking in different ways (Sommerfeld et al., 2000; Chen et al., 2005), which may also impact how these receptors interact with p75^*NTR*^. In this study, we have demonstrated that full-length p75^*NTR*^ interacts with TrkB, and that they are internalized together into early endosomes.

### Regulation of Neuronal Survival

The cellular consequences of p75^*NTR*^ actions are strongly dependent on the cell context and may be determined by which co-receptors are engaged as well as which intracellular adapter proteins can be recruited to signal (Nykjaer et al., 2005). Many studies in the literature have investigated the role of p75^*NTR*^ with respect to neuronal survival, with some studies showing pro-survival effects and some showing apoptotic induction, especially after injury. We have demonstrated that some pro-survival effects of p75^*NTR*^ may be due to facilitation of TrkB trafficking and signaling in response to BDNF, apart from direct activation of survival signaling by p75^*NTR*^. In this study we showed that phosphorylation of Akt by BDNF, which is induced by TrkB signaling, was attenuated in the absence of p75^*NTR*^. Since the PI_3_K-Akt signaling pathway mediates neuronal survival in many paradigms, we investigated whether the lack of p75^*NTR*^ compromised neuronal survival. Although basal survival of hippocampal neurons was not impaired in the absence of p75^*NTR*^, a trophic deprivation assay with the depletion of insulin from the media elicited neuronal death, which was rescued by BDNF in WT neurons but not p75^*NTR*–/–^ neurons.

In sum, we have demonstrated that BDNF induces the association of TrkB with p75^*NTR*^ following TrkB phosphorylation and internalization of the receptors to early endosomes. The absence of p75^*NTR*^ attenuated activation of Akt, but not Erk, and prevented the ability of BDNF to rescue hippocampal neurons from trophic deprivation, suggesting that p75^*NTR*^ may facilitate the ability of TrkB to activate specific downstream signaling pathways to regulate neuronal survival and function.

## Data Availability Statement

The datasets generated for this study are available on request to the corresponding author.

## Ethics Statement

The animal study was reviewed and approved by the Rutgers IACUC.

## Author Contributions

JZ, LM, and MV performed the experiments. JZ, LM, and WF analyzed the data. WF wrote the manuscript.

## Conflict of Interest

The authors declare that the research was conducted in the absence of any commercial or financial relationships that could be construed as a potential conflict of interest.
